# Test–Retest Reliability of Isokinetic Strength in Lower Limbs under Single and Dual Task Conditions in Women with Fibromyalgia

**DOI:** 10.3390/jcm13051288

**Published:** 2024-02-24

**Authors:** Mari Carmen Gomez-Alvaro, Juan Luis Leon-Llamas, Maria Melo-Alonso, Santos Villafaina, Francisco Javier Domínguez-Muñoz, Narcis Gusi

**Affiliations:** 1 Physical Activity and Quality of Life Research Group (AFYCAV), Faculty of Sport Sciences, University of Extremadura, 10003 Cáceres, Spain; maricarmengomezal@unex.es (M.C.G.-A.); leonllamas@unex.es (J.L.L.-L.); mmeloa@unex.es (M.M.-A.); svillafaina@unex.es (S.V.); fjdominguez@unex.es (F.J.D.-M.); 2 Institute for Research and Innovation in Sport Sciences, University of Extremadura, 10003 Cáceres, Spain

**Keywords:** isokinetic dynamometry, muscle strength, muscle strength dynamometer, chronic pain, fibromyalgia, assessment, activities of daily living, mobility

## Abstract

**Background**: Previous research has established good test–retest reliability for isokinetic dynamometry in fibromyalgia. However, the reliability of this test under dual-task conditions has not been investigated in fibromyalgia. **Methods**: A total of 10 women with fibromyalgia participated in this study. Participants completed the concentric/concentric test. The dual-task condition involved subtracting two by two while performing the test. **Results**: Reliability analysis under the single condition showed “poor” to “excellent” values for maximum peak torque in knee extension and “moderate” to “excellent” values for average. “Poor” to “excellent” reliability values were found in knee flexion for the maximum and average. Dual-task condition in knee extension ranged from “moderate” to “excellent” for maximum and average values, and in knee flexion, it ranged from “poor” to “excellent” for maximum value and from “moderate” to “excellent” for average value. **Conclusions**: Isokinetic dynamometry demonstrated sufficient reliability for measuring strength in knee extension maximum and average during single-task and dual-task conditions, along with knee flexion dual-task average in fibromyalgia. For knee flexion single-task maximum and average and knee flexion dual-task maximum, we did not obtain sufficiently reliable measurements. Only the concentric/concentric test has been studied, and future studies with a larger sample size are needed in order to generalize the results.

## 1. Introduction

Fibromyalgia (FM) affects 0.2–6.6% of the general population and predominantly women over 50 years [1]. Individuals with FM, in addition to experiencing widespread chronic pain, may endure other symptoms such as muscle stiffness, persistent fatigue, disrupted sleep patterns, and an increased susceptibility to mood disorders, such as anxiety and depression [2]. This symptomatology can result in both cognitive impairment [3,4] and physical limitations [5,6,7]. Regarding physical limitations, there is generally a reduction in muscle strength in both the upper and lower limbs [8]. In the lower limbs, people with FM exhibit lower muscle strength, fatigue resistance, and static endurance [6,9,10,11,12]. Specifically, muscle strength in the quadriceps is diminished, probably due to reduced muscle mass [13,14] or reduced muscle strength per cross-sectional area [15,16]. Therefore, muscle function is globally impaired in FM people [6], as is their overall functionality [12]. In this context, peak torque of the lower limbs has been studied in individuals with FM, along with its potential association with the risk of falls [17].

As a result of their symptoms, FM people often experience a decline in their quality of life [18], partly due to the challenges they face in performing their activities of daily living [19]. It is rare to perform a task simultaneously every day. Instead, daily living activities often involve performing two tasks simultaneously, such as walking and talking or climbing stairs while thinking about something, a concept known as dual-task (DT) [20,21]. When attention is distributed between two simultaneous activities, a decrease in performance on both tasks may be experienced, in contrast to when they are performed individually [22], and healthy nervous systems have been shown to have limitations in information processing, which affects the ability to multitask [23]. In individuals with neurodegenerative diseases, these limitations may manifest themselves more markedly and significantly affect the performance of their daily tasks [24]. The relative change in performance associated with dual-task performance is known as dual-task interference, and it has been shown that individuals with neurological deficits may be particularly susceptible to this interference, as increased attentional demands to control motor performance reduce the availability of attentional resources to perform simultaneous secondary tasks [24]. In this way, it has been noted that individuals with FM exhibit more significant interference when performing two tasks simultaneously compared to healthy individuals, consequently affecting their performance in one or both tasks [25,26,27,28,29].

Several studies have already focused on assessing the reliability of various tests and measures under DT conditions [30,31,32]. This is crucial, since evaluating these activities taking into account not only physical demands but also cognitive factors such as attention and executive functions makes them more representative of real-life demands [20,21,33]. In this context, reliable tests to assess muscle strength are fundamental. Reliability refers to the consistency of the results when the same methodology is applied under similar conditions [34]. One of the reliable methods for measuring lower limb muscle strength in FM is isokinetic dynamometry [35], which is one of the most commonly employed tools in this context [6,8,9,11,12,14,15,16,36,37,38]. This method enables the measurement of dynamic muscle strength through electromechanical devices that maintain a constant velocity of movement while keeping the resistance consistent throughout the range of motion [39,40]. 

To the best of our knowledge, no previous study has evaluated the reliability of this test in FM people during DT conditions. Therefore, the present study aimed to analyze the test–retest reliability of the maximum (Max) and average (Avg) peak torque knee flexion and extension under single-task (ST) and DT conditions. Thus, we hypothesized that good test–retest reliability would be achieved in isokinetic dynamometry, both in ST and DT conditions, in line with findings from previous studies [35]. 

## 2. Materials and Methods

### 2.1. Participants

A sample size of 10 Caucasian women with FM from Caceres (Spain) with two observations per subject achieved 81% power to detect an intraclass correlation of 0.95 under the alternative hypothesis when the intraclass correlation under the null hypothesis is 0.75 using an F-test with a significance level of 0.05. The convenience sample was recruited from a local association to participate in this test–retest study. The PASS software (version 11.0; PASS; Kaysville, UH, USA) was employed to calculate the power and the sample size.

The inclusion criteria were set as follows: (a) women aged 30–75 years; (b) capable of effective communication with the research staff; (c) having read, comprehended, and signed the informed consent form; (d) confirmed diagnosis of fibromyalgia by a rheumatologist according to the American College of Rheumatology criteria [41]. 

The exclusion criteria were: (a) presence of psychiatric or neurological disorders; (b) receiving pharmacological treatment for depression or anxiety; (c) abuse or dependency on substances; (d) inability to engage in physical exertion due to contraindications; (e) injury to the leg affecting the flexion and extension of the knee; and (f) pregnancy status.

The present study was approved by the Research Ethics Committee of the University of Extremadura (approval reference: 192/2021). All participants were informed about the procedures and gave their written informed consent following the updated Declaration of Helsinki.

### 2.2. Instruments and Procedure

Participants were asked about years with FM, their level of education—primary education or elementary school graduate, professional training or secondary education, university studies—and the Spanish version of the Revised Fibromyalgia Impact Questionnaire (FIQR) was administered [42]. This questionnaire is validated for people with FM and consists of 21 items, with a maximum score of 100 indicating the worst outcomes [43]. In addition, the Spanish version of the International Physical Activity Questionnaire (IPAQ) was used to assess physical activity and inactivity in this study, reporting the physical activity classification based on the different activities developed at work, for getting around, during leisure time, and for household tasks [44]. The Montreal Cognitive Assessment (MoCA) was also employed. MoCA is a concise cognitive screening tool that assesses various cognitive abilities, including attention, concentration, executive functions, memory, language, visual-constructional skills, calculation, and orientation [45]. MoCA has been found to be more sensitive than MMSE in individuals with FM [46]. Furthermore, a cut-off score of 23 out of 30 has been established to enhance diagnostic accuracy, effectively reducing the false positive rate [47]. Finally, age and anthropometric measurements were acquired using a Tanita Body Composition Analyzer BC-418 MA (Tanita Corp., Tokyo, Japan) and a stadiometer SECA 769 column scale (SECA Corp, Hanover, MD, USA).

In line with previous research addressing the reliability of isokinetic parameters in FM, the same procedure was performed on two different occasions, specifically on days 1 and 2, with an interval of 7 days between them [35]. The testing exclusively involved the dominant leg, and the evaluation of the weight of the limb and gravity adjustments were conducted using dynamometric software.

The test was performed using the isokinetic dynamometer (Multi-Joint 3, Biodex Medical Systems, Inc., Shirley, NY, USA). The test consists of the consecutive performance of five concentric actions, specifically for knee flexion and extension, constituting the concentric/concentric test [39,48]. These actions are carried out without interruption at a velocity of 60°/s within a range of motion from 0 to 90 degrees, where 0 degrees corresponds to full extension and 90 degrees represents flexion [48]. The setup and positioning instructions for knee extension/flexion provided by the manufacturer were followed. Each participant was attached to the dynamometer seat, aligning their knee axis with the dynamometer’s axis. The dynamometer was kept untilted at 0°, with the seat and isokinetic dynamometer positioned at 90° and the seatback tilt set at 85°. The dynamometer software was used to assess the weight of the leg being tested. In addition, to ensure the consistency of the measurements, a calibration of the system was performed before starting the test, following the indications provided in the application/operation manual provided by the manufacturer.

The testing protocol includes several steps: First, a general warm-up comprising 10 squats without load was performed. Following the general warm-up, participants engaged in a familiarization phase or specific warm-up, during which they completed 10 consecutive knee flexion and extension repetitions at a speed of 120°/s. There was a 1 min rest period before starting the final test.

During the DT conditions, participants simultaneously performed the concentric/concentric test described above and, at the same time, a cognitive task. Specifically, the cognitive task required participants to subtract numbers two by two from a value greater than 100, which was chosen at random, being odd or even [30].

### 2.3. Statistical Analysis

Statistical analysis was conducted using the Statistical Package for the Social Sciences software (SPSS, version 24.0; IBM Corp., Armonk, NY, USA) to analyze the data. The mean and standard deviation of each variable were calculated to characterize the sample, and the Max and Avg peak torques in knee extension and flexion were calculated for the test and retest. The 3.1 (two-way mixed effects, single rater/measurement) model was used to estimate the intraclass correlation coefficient (ICC) and the 95% confidence intervals of the peak torque knee extension and flexion under single and dual-task conditions, following the recommendations provided by Weir [49] and Koo [50]. The ICC classification was interpreted according to the guidelines proposed by Koo [50], where ICC values lower than 0.50 indicate “poor” reliability, values between 0.50 and 0.75 indicate “moderate” reliability, values between 0.75 and 0.90 indicate “good” reliability, and values higher than 0.90 indicate “excellent” reliability.

The absolute reliability was extracted by calculating the standard error of measurements (SEM) using the following formula:

$$\mathrm{SEM}=\mathrm{SD}\times \sqrt{1-\mathrm{ICC}}$$

where SD is the mean SD of the 5 repetitions in the test and retest for the average peak torque or the mean SD of the highest repetition in the test and retest for the Max peak torque.

To demonstrate if the change score in individual performance represents real and reliable change, the smallest real difference (SRD) was obtained using the following formula:

$$\mathrm{SRD}=1.96\times \mathrm{SEM}\times \sqrt{2}$$

Both SEM and SRD were converted into percentages. The results of these percentages were calculated according to the following formula, SEM% or SRD%=(SEM or SRD/mean)×100, where the mean is the average of the test and retest.

To identify the level of agreement between the test and retest, Bland–Altman plots were performed for Max peak torque and Avg peak torque under single and dual-task conditions [51]. 

Moreover, to understand whether there was a relationship between test performance, the impact of the FM, and years of FM symptomatology, Spearman’s Rho correlation analysis was performed between the peak and average value of the test in single and dual-task conditions and the FIQR values and years of FM symptomatology. The statistical significance was set at the *p* ≤ 0.05 level.

## 3. Results

The main characteristics of the women are shown in Table 1. The results showed that the participants had an age of 48.50 (7.79), ranging from 36 to 62 years, indicating a moderate severity impact of the disease [52]. They suffered 12.44 (6.37) years of the disease since diagnosis and did not exhibit cognitive impairment [47]. Regarding their educational level, 4 participants completed primary or elementary school, 4 had professional training or secondary education, and 2 had university studies. Moreover, eight women were identified as being moderately physically active, one woman as being intensely physically active, and one woman as being low in physical activity, according to the values reported in the IPAQ questionnaire. Regarding medication intake, 10 individuals were taking NSAIDs, and 2 were taking opioids.

The descriptive data of the test and retest for the Max peak torque and the Avg peak torque of the five repetitions of knee extension and flexion under single and DT conditions are shown in Table 2. First, reliability analysis showed that the women with FM reported under the single condition “poor” to “excellent” reliability values for Max peak torque in knee extension and “moderate” to “excellent” reliability values for Avg peak torque. However, “poor” to “excellent” reliability values were found in knee flexion for the Max and Avg peak torque. Secondly, reliability values for the dual-task condition in knee extension ranged from “moderate” to “excellent” for Max and Avg values, and in knee flexion ranged from “poor” to “excellent” for Max value and from “moderate” to “excellent” for Avg value. Finally, SEM and SRD values were also reported (see Table 2).

**Figure 1:**
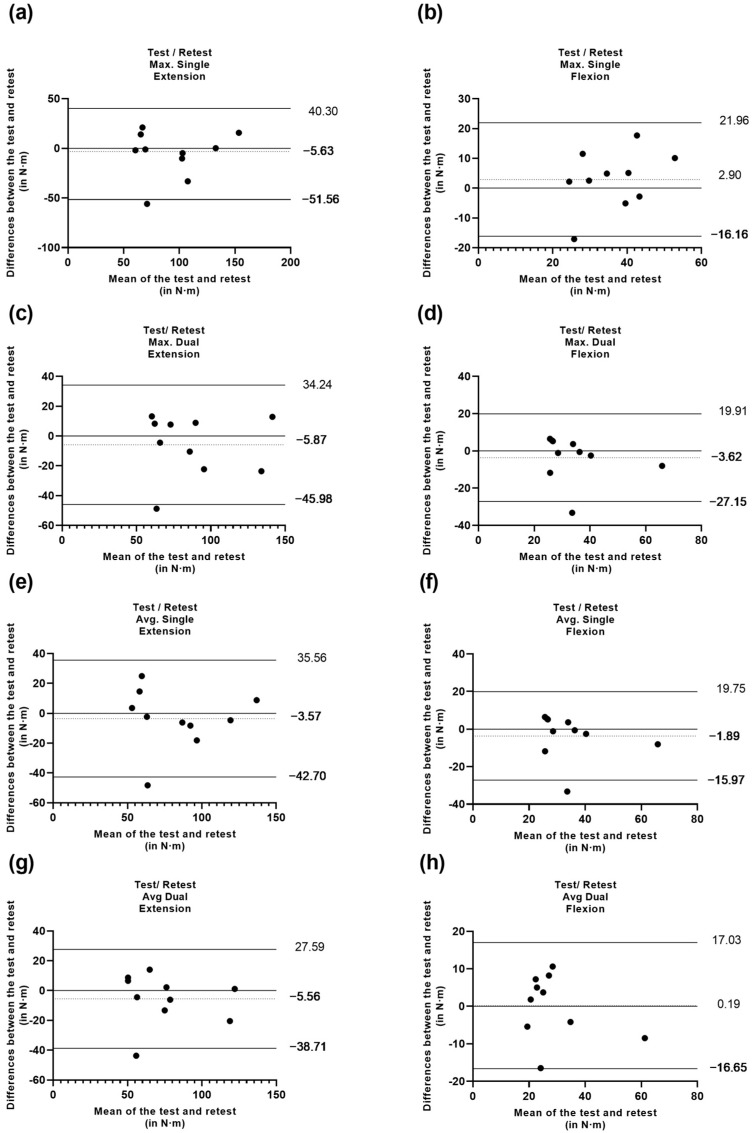
(**a**) differences between test and retest vs. the mean of the two measurements of peak torque maximum in extension under the single condition; (**b**) differences between test and retest vs. the mean of the two measurements of peak torque maximum in flexion under the single condition; (**c**) differences between test and retest vs. the mean of the two measurements of peak torque maximum in extension under the dual-task condition; (**d**) differences between test and retest vs. the mean of the two measurements of peak torque maximum in flexion under the dual-task condition; (**e**) differences between test and retest vs. the mean of the two measurements of peak torque average in extension under the single condition; (**f**) differences between test and retest vs. the mean of the two measurements of peak torque average in flexion under the single condition; (**g**) differences between test and retest vs. the mean of the two measurements of peak torque average in extension under the dual-task condition; (**h**) differences between test and retest vs. the mean of the two measurements of peak torque average in flexion under the dual-task condition.

To obtain a more comprehensive analysis, the level of agreement for the test and retest under single and DT conditions was analyzed through the Bland–Altman plots depicted in Figure 1.

Finally, the correlation analysis between the performance obtained in the test through the peak and average values in flexion and extension in single and dual-task conditions was not significantly related to the impact of the disease nor the years of FM symptomatology (see Supplementary Material Table S1). All values showed a *p*-value > 0.05.

## 4. Discussion

The main objective of this study was to establish the test–retest reliability of isokinetic dynamometry during DT conditions in FM people. To our knowledge, no previous study has reported the reliability of isokinetic knee measurements in FM patients under DT conditions. The findings support the application of DT conditions as a reliable tool for assessing physical fitness in real-life conditions. However, some measures did not yield values that were sufficiently high (>0.8) [53], and confidence intervals exhibited significant variability in the majority of cases. The ICC values for peak torque measurements in the present study indicate that the reliability of the isokinetic dynamometry, under DT conditions in both knee flexion and extension, ranged from “moderate” to “good” for maximum and average values. In particular, the test could be considered sufficiently reliable for measuring strength in knee extension maximum and average during both ST and DT conditions, as well as knee flexion DT average. However, for the other measurements—specifically, knee flexion ST maximum and average and knee flexion DT maximum—we did not obtain sufficiently reliable measurements.

By analyzing the data obtained in the DT condition to show the variability of the results in the isokinetic test due to measurement error, our study reported percentages to quantify the accuracy of the scores. In this sense, there was a 12.37 and 11.64% probability (with a 95% confidence interval) that a repeated measurement in the isokinetic dynamometry test would show a difference of 10.78 N·m and 8.71 N·m from the initial knee extension score for the maximum and average values, respectively. Similarly, there was a 19.35 and 15.70% probability that a repeated measure in the isokinetic dynamometry test showed a difference of 6.64 N·m and 4.49 N·m in knee flexion score for the maximum and average values, respectively. Considering that the SEM provides a range of scores in which the actual score of a specific test is expected to lie, achieving the lowest possible SEM values is crucial to ensuring higher reliability [53]. 

In terms of the minimum amount of change that must be observed in a score for it to be considered a real or significant change beyond measurement error, our study also reported the %SRD. In this line, in the scores obtained in the DT condition, the %SRD ranged between 34.28 and 53.65% in its maximum values for extension and flexion, respectively, and between 32.27 and 43.52% in its average values for extension and flexion. The SRD results suggest that improvements greater than 29.87 N·m at peak and 24.14 N·m at average peak in knee extension, as well as 18.41 N·m and 12.44 N·m in knee flexion, indicate changes that are not attributable to measurement error or subject variability and are relevant information for researchers and exercise and health professionals. Although this measure has been previously reported in other populations [54,55], its application in isokinetic dynamometry under DT conditions in women with FM has not been previously investigated. 

Our reliability results differ slightly from those previously reported in FM [35], which only measured the performance of this isokinetic test in the ST condition. Furthermore, if we compare the values obtained in our study for the DT condition, they differ slightly from those of the study mentioned above. However, it is essential to note that the study conducted by Adsuar, Olivares, del Pozo-Cruz, Parraca, and Gusi [35] did not report mean peak torque values and analyzed the maximum of three repetitions. In contrast, our analysis was based on a maximum of five repetitions and considered the average value of the five repetitions as a more ecological and representative measure of the participants’ performance. 

Given that the results obtained in our study have shown variability in ICC values, different factors may have influenced this aspect.

We must emphasize that we have a small sample and that the symptoms of FM can change. Although in our study no significant relationships were found between the performance obtained in the test, the impact of the disease, and the years with FM in the different conditions, taking into account the pain at baseline, individual variability in the pain threshold or muscle fatigue experienced during the isokinetic test may have altered the participants’ performance. In this sense, future studies must contemplate these aspects to obtain more precise information and determine whether the variability in the results may be due to these factors [56]. It would be desirable to replicate this study with a larger sample to more accurately determine the reliability of isokinetic dynamometry for measuring lower limb strength in ST [35] and DT conditions in women with FM.

Our results could have yielded lower values than other investigations due to the type of warm-up performed. In this line, Bishop [57] suggests that a warm-up performed at ~40–60% VO2 max for 5 to 10 min, followed by a 5 min recovery, will improve short-term performance. However, to avoid bias in our results, we followed the same warm-up protocol specified in our study for the test and retest. More research is needed to understand the ergogenic effects of warm-up on short-term performance, as it remains controversial [57].

Following a detailed analysis, significant variability in confidence intervals has been observed in both the ST and DT conditions, particularly in knee flexion. The ICC for maximum knee flexion peak torque was (0.72, 95% CI −0.13–0.93) and (0.77 95% CI 0.09–0.94) for average peak torque in the ST condition compared to (0.90 95% CI 0.03–0.94) and (0.91 95% CI 0.51–0.98) in the DT condition. This difference in results could be attributed to the possibility that individuals with FM might pay more attention to pain awareness during flexion, depending on the pain level they experience at that moment or the fear of experiencing that pain. However, an interesting issue was that the reliability value slightly increased in the DT condition. This could be explained by the potential for individuals to divert their focus to the cognitive task effectively and thus not pay attention to the level of pain they may be experiencing at the time. In this regard, a negative relationship has been observed between pain sensitivity and functional capacity in specific tests [58]. Individuals with FM struggle to regulate their emotional states, facing difficulties in concentration and task performance during negative mood states, which has a significant impact on pain severity and disability severity [59]. In addition, pain-related fear has been associated with increased pain, decreased tolerance to physical performance, reduced cognitive performance speed, increased sensitivity to painful points, and increased interference with other attention-demanding tasks [60]. Therefore, greater attention to pain, and its variability, could contribute to less reliable test results, highlighting the potential benefits of introducing a simultaneous cognitive task (DT) to assess lower limb strength in FM. 

In this context, de Gier, Peters, and Vlaeyen [60] have found that tolerance to physical performance increases when distraction is introduced by a simultaneous cognitive task. In chronic pain patients, Rode et al. [61] have also demonstrated that the inclusion of a distracting task significantly enhances physical performance, elevating the initial tolerance level to a comparable level to that of pain-free controls. Our results could support the hypothesis that, although pain captures attention, it can be interrupted by employing another distracting task. This makes sense considering that in FM, pain has been observed to interfere with attention, involving the utilization of neural resources engaged in both processes [62]. In our study, the results were apparently slightly better in DT than when the test was performed without the cognitive task. Nevertheless, it is essential to consider that the interpretation of these results may be influenced by various pain-related characteristics (intensity, novelty, and predictability of threatening stimuli, as well as the tendency to catastrophize pain and heightened somatic awareness) and environmental factors (distractibility of pain and its emotional content and arousal properties) [43].

The assessment of DT conditions in fitness tests has been evaluated and proven reliable, aligning more closely with real-life conditions. It requires heightened attentional resources and engagement of executive functions [33], which are impaired in individuals with FM [63,64,65]. In this sense, the reliability of different tests such as the chair stand test [30], 10 m walking test [31], Timed Up and Go test (TUG) [31], arm curl test [30], and 3 m walking backward test (3MBWT) [32] have been investigated under DT conditions among people with FM. The findings from our study can complement previous research, expanding the range of reliable tests for assessing performance in DT situations in individuals with FM. Moreover, it presents a relevant interest in assessing physical performance since people with FM show lower values in peak torque than healthy people [17]. Furthermore, there is an association between a high prevalence of falls with lower functional performance and lower limb strength [17]. Therefore, it would be beneficial to use tests that consider DT performance to continue studying falls in FM, as may occur during activities of daily living.

The present study has some limitations. First, the sample was relatively small, and comprised solely of women, preventing the generalization of the results to the male population. However, it is essential to note that the morphological differences in the musculature can generate significant alterations in the mechanical properties of the muscles depending on the sex [66]. In this sense, future studies should consider this aspect to elucidate possible sex-related differences. Secondly, the age of the sample was between 30 and 75, a wide range that could influence both physical and cognitive performance. Third, individual variability in pain threshold and muscle fatigue experienced during the isokinetic test could have altered the performance of the participants. Fourth, the absence of specific neurological assessments to exclude co-existing neuropathies or other neurological conditions that might impact muscle strength testing complicates the interpretation of results solely attributable to FM. Fifth, only the concentric/concentric test was performed, so the results cannot be compared with other studies using different tests. Therefore, further studies exploring alternative tests are needed.

## 5. Conclusions

Isokinetic dynamometry demonstrated sufficient reliability for measuring strength in knee extension maximum and average during both single-task and dual-task conditions, as well as knee flexion dual-task average. However, for knee flexion single-task maximum and average and knee flexion dual-task maximum, we did not obtain sufficiently reliable measurements. Only the concentric/concentric test has been studied, and future studies with a larger sample size are needed in order to generalize the results.

## Figures and Tables

**Table 1:** Descriptive characteristics of the participants.

Variables (N = 10)	Mean (SD)
Age (years)	48.50 (7.79)
Height (cm)	158.40 (0.07)
Weight (kg)	74.26 (18.55)
Years with FM	12.44 (6.37)
FIQR	54.30 (24.28)
MoCA	25.80 (1.69)
Educational level	
Primary education/elementary school	4
Professional training/secondary education	4
University studies	2
IPAQ Classification	
Intense physical activity	1
Moderate physical activity	8
Low physical activity	1
Drug classes	
NSAIDs	10
Opioids	2

Abbreviations: N, sample; SD, standard deviation; FM, fibromyalgia; FIQR, Fibromyalgia Impact Questionnaire Revised; MoCA, Montreal Cognitive Assessment, IPAQ, International Physical Activity Questionnaire; NSAIDs, nonsteroidal anti-inflammatory drugs.

**Table 2:** Maximum and average peak torque reliability under single and dual-task conditions.

Variables		Test Mean (SD)	Retest Mean (SD)	ICC (95% CI)	SEM	%SEM	SRD	%SRD
Single-task								
Knee extension (N·m)	Max	96.09 (32.79)	90.46 (35.16)	0.87 (0.46–0.97)	12.25	13.13	33.95	36.40
	Avg	84.72 (30.44)	81.15 (30.07)	0.88 (0.51–0.97)	10.48	12.64	29.05	35.03
Knee Flexion (N·m)	Max	34.67 (8.60)	37.57 (11.89)	0.72 (−0.13–0.93)	5.42	15.01	15.03	41.60
	Avg	30.64 (9.59)	32.53 (11.56)	0.77 (0.09–0.94)	5.07	16.06	14.06	44.51
Dual-task								
Knee extension (N·m)	Max	90.08 (31.06)	84.21 (31.16)	0.88 (0.51–0.97)	10.78	12.37	29.87	34.28
	Avg	77.58 (28.48)	72.02 (26.59)	0.90 (0.58–0.97)	8.71	11.64	24.14	32.27
Knee flexion (N·m)	Max	36.12 (14.70)	32.50 (12.41)	0.76 (0.03–0.94)	6.64	19.35	18.41	53.65
	Avg	28.50 (14.23)	28.69 (11.69)	0.88 (0.51–0.98)	4.49	15.70	12.44	43.52

Abbreviations: N·m, Newton meter; Max, maximum; Avg, average; SD, standard deviation; ICC, intraclass correlation coefficient; CI, confidence interval; SEM, standard error of measurement; SRD, smallest real difference.

## Data Availability

The data will not be shown publicly, as patients will give their consent for the information to be kept confidential.

## Supplementary Materials

The following supporting information can be downloaded at: https://www.mdpi.com/article/10.3390/jcm13051288/s1, Table S1. Correlation between the performance obtained in the isokinetic test under single and dual-task conditions in test and retest, FIQR and years of fibromyalgia symptomatology.
