# Supplementary material for: Test–Retest Reliability of Isokinetic Strength in Lower Limbs under Single and Dual Task Conditions in Women with Fibromyalgia
Source: J Clin Med. 2024 Feb 24;13(5):1288. doi: 10.3390/jcm13051288 (PMC10932145; doi:10.3390/jcm13051288)
Supplement: Supplementary file 1 [file jcm-13-01288-s001.zip › jcm-2824163-supplementary.pdf]

SUPLEMENTARY MATERIAL

Table S1. Correlation between the performance obtained in the isokinetic test under single and dual-task conditions in test and retest, FIQR and years of fibromyalgia symptomatology.

Variables			FIQR	Years with Fibromyalgia
Single	Knee Extension Max. (test)	Correlation coefficient	0.091	-0.185
		p-value	0.803	0.634
	Knee Extension Max. (retest)	Correlation coefficient	0.406	-0.101
		p-value	0.244	0.796
	Knee Extension Avg. (test)	Correlation coefficient	0.067	-0.126
		p-value	0.855	0.747
	Knee Extension Avg. (retest)	Correlation coefficient	0.418	-0.067
		p-value	0.229	0.864
	Knee Flexion Max. (test)	Correlation coefficient	-0.030	-0.521
		p-value	0.934	0.150
	Knee Flexion Max. (retest)	Correlation coefficient	0.164	-0.148
		p-value	0.650	0.705
	Knee Flexion Avg. (test)	Correlation coefficient	-0.006	-0.588
		p-value	0.987	0.096
	Knee Flexion Avg. (retest)	Correlation coefficient	0.212	-0.252
		p-value	0.556	0.513
Dual-Task	Knee Extension Max. (test)	Correlation coefficient	0.285	-0.042
		p-value	0.425	0.915
	Knee Extension Max. (retest)	Correlation coefficient	0.345	-0.092
		p-value	0.328	0.813
	Knee Extension Avg. (test)	Correlation coefficient	0.248	-0.067
		p-value	0.489	0.864
	Knee Extension Avg. (retest)	Correlation coefficient	0.491	0.067
		p-value	0.150	0.864
	Knee Flexion Max. (test)	Correlation coefficient	0.127	-0.185
		p-value	0.726	0.634
	Knee Flexion Max. (retest)	Correlation coefficient	0.321	-0.218
		p-value	0.365	0.572
	Knee Flexion Avg. (test)	Correlation coefficient	0.248	-0.076
		p-value	0.489	0.847
	Knee Flexion Avg. (retest)	Correlation coefficient	0.067	-0.210
		p-value	0.855	0.587

Abbreviations: FIQR, fibromyalgia impact questionnaire revised; Max, Maximum; Avg, Average.
